# Performance of a Nonlinear Magnetic Handheld Probe for Intraoperative Sentinel Lymph Node Detection: A Phantom Study

**DOI:** 10.1245/s10434-023-14166-z

**Published:** 2023-09-04

**Authors:** Eliane R. Nieuwenhuis, Nida Mir, Melissa M. Horstman-van de Loosdrecht, Antoi P. W. Meeuwis, Maarten G. J. de Bakker, Tom W. J. Scheenen, Lejla Alic

**Affiliations:** 1 Magnetic Detection and Imaging Group, Technical Medical Centre, University of Twente, Enschede, The Netherlands; 2 Department of Medical Imaging, Radboud University Medical Center, Nijmegen, The Netherlands

**Keywords:** Magnetic handheld probe, Gamma probe, Sentinel lymph node, Detection, Superparamagnetic iron oxide nanoparticle

## Abstract

**Objective:**

This study investigates the performance of the DiffMag handheld probe (nonlinear magnetometry), to be used for sentinel lymph node detection. Furthermore, the performance of DiffMag is compared with a gamma probe and a first-order magnetometer (Sentimag^®^, linear magnetometry).

**Methods:**

The performance of all three probes was evaluated based on longitudinal distance, transverse distance, and resolving power for two tracer volumes. A phantom was developed to investigate the performance of the probes for a clinically relevant situation in the floor of the mouth (FOM).

**Results:**

Considering the longitudinal distance, both DiffMag handheld and Sentimag^®^ probe had comparable performance, while the gamma probe was able to detect at least a factor of 10 deeper. Transverse distances of 13, 11, and 51 mm were measured for the small tracer volume by the DiffMag handheld, Sentimag^®^, and the gamma probe, respectively. For the large tracer volume this was 21, 18, and 55 mm, respectively. The full width at half maximum, at 7 mm probe height from the phantom surface, was 14, 12, and 18 mm for the small tracer volume and 15, 18, and 25 mm for the large tracer volume with the DiffMag handheld, Sentimag^®^, and gamma probe, respectively.

**Conclusions:**

With a high resolving power but limited longitudinal distance, the DiffMag handheld probe seems suitable for detecting SLNs which are in close proximity to the primary tumor. In this study, comparable results were shown using linear magnetometry. The gamma probe reached 10 times deeper, but has a lower resolving power compared with the DiffMag handheld probe.

Most solid tumors usually spread via the lymphatic vessels to the locoregional lymph nodes (LNs).^1,2^ Since the presence of metastasis is a key factor in the patient’s prognosis and subsequent treatment planning, the lymphatic status must be accurately determined.^3^ A sentinel lymph node biopsy (SLNB) is introduced as a minimally invasive procedure to identify and remove the first draining LNs, referred to as sentinel lymph nodes (SLNs).^3,4^ The SLNB is often enabled by a peritumoral tracer injection. This allows the tracer, via the lymphatic vessels, to accumulate in the SLN(s) and enables intraoperative detection by a handheld probe. This same path is followed by potential metastases, therefore the SLN has the highest chance of containing metastasis and is of interest in diagnosing the tumor stage.

The conventional SLNB procedure uses a radioactive tracer and a handheld gamma probe for intraoperative SLN detection. Despite the effectiveness of this procedure,^5,6^ alternatives are being considered due to limited production sites, complicated logistics, and regulations. A recently introduced alternative utilizes a magnetic probe and a magnetic tracer consisting of superparamagnetic nanoparticles (SPIONs). In breast cancer, this magnetic procedure has been shown to be non-inferior to the procedure using radioisotopes.^7,8^ However, since the first-order magnetometer (currently in clinical use) relies on linear magnetic detection, it captures all magnetic signals including those of the surgical instruments and the diamagnetic human body.^9^ As a result, the SLNB procedure requires non-metallic instruments as well as frequent balancing of the magnetic probe.^10,11^ Although the balancing is relatively simple and fast, it still interrupts the flow of the SLNB procedure as it needs frequent repetitions. Hence, a patented nonlinear magnetic detection principle, differential magnetometry referred to as DiffMag, has been presented by the University of Twente to overcome these drawbacks.^12^ A magnetic handheld probe (DiffMag handheld probe), utilizes nonlinear SPION characteristics by augmenting an alternating excitation magnetic field with a DC offset, which results in a signal characteristic solely for the magnetization of SPIONs.^9,13^

Clinical and consequent technical requirements for a handheld probe are prioritized differently depending on the location of the SLN with respect to the primary tumor. In cases where the SLNs are located deeper and at distance to the injection spot, the longitudinal distance becomes more relevant, for example in breast cancer.^14^ If the SLN is located near the primary tumor and hence the injection spot, a high resolving power is warranted. For example in the floor of the mouth (FOM), a so-called shine-through phenomenon makes it difficult to distinguish the SLN close to the injection spot using the conventional technique.^15^

To gain insight into the current technical performance of the DiffMag handheld probe relevant for application in the FOM, a phantom study was conducted that mimics one injection spot at the primary tumor site and a tracer volume accumulated in an SLN. The proposed sample volumes are based on the international guidelines and clinical studies evaluating the injection spot^16–18^ and the accumulation of tracer in an SLN.^19–21^ The phantom is used to determine the longitudinal distance, the transverse distance, and the resolving power between two adjacent SLNs. The performance of the DiffMag handheld probe was tested and compared with a first-order magnetometer and a gamma probe.

## Methods

A phantom was used to assess longitudinal distance, transverse distance and resolving power for two sample volumes by three handheld probes (two magnetic and one gamma probe). This section includes a description of the phantom, the probes and tracers used, and the experiments performed.

### Phantom

A Delrin^®^ (polyoxymethylene) phantom (University of Twente, the Netherlands) is non-magnetic and non-reactive to all the tracers used. As illustrated schematically in Appendix A, the phantom consists of 13 accurately milled columnar triplet pits. The SolidWorks^®^ (SOLIDWORS 2021, Dassault Systemes SolidWorks Corporation) design file of the phantom is available online.^22^ Each triplet contained two small pits (capacity 5 µl) and one large pit (capacity 500 µl). The small pits represent tracer accumulation in an SLN, while the large pit represents one injection (out of four) at a primary tumor site. The distances between the pits vary between 3 and 25 mm to mimic a range of realistic distances between SLNs and injection sites in the FOM.^23^ For the experiments, only one column at a time was filled with one or two adjacent pits.

### Probes

A DiffMag handheld probe (University of Twente, The Netherlands) was used for the detection of magnetic tracer based on a patented nonlinear magnetic detection principle, differential magnetometry (DiffMag).^12^ The probe tip has a diameter of 22 mm. It was used with in-house software, with an excitation frequency of 2.5 kHz.

A Sentimag^®^ probe (Endomagnetics Ltd., UK) was used for magnetic detection of magnetic tracer based on its linear magnetization properties. The probe tip has a diameter of 18 mm. Measurements were performed at sensitivity level 1. Prior to each acquisition, the probe was balanced in air at a distance of at least 30 mm from the magnetic tracer. A balancing procedure takes about 7 s.^24^

The Europrobe 3.2 (CdTe SOE311, EuroMedical Instruments, France) is a gamma probe consisting of a solid-state ionization detector where the gamma-ray photons create electron-pit pairs and therefore induce signals on electrodes, creating an electrical current. The probe has a 60% detector efficiency for ^99m^Tc (20–170 keV). The probe is angled, with the head diameter being 11 mm. Detection time varied between 2 s (dose used as injection spot), and 10 s (dose used as LN).

### Tracers

The open-source samples with the volumes used are summarized in Table 1. A clinically available magnetic tracer, Magtrace^®^ [28 mg iron/ml] (Endomagnetics Ltd., UK), was used in conjunction with the two magnetic probes. For the volume representing an SLN, Magtrace^®^ was undiluted, whereas for the injection spot, a volume of 100 µl Magtrace^®^ was diluted with 300 µl water. ^99m^Tc pertechnetate, referred to as ^99m^Tc tracer, was used undiluted for the gamma probe measurements.

**Table 1:** Volumes of Magtrace^®^ and ^99m^Tc-tracer used in experiments representing tracer accumulation in an SLN and injection spot

	Sample volume, µl	Amount	Representing
Magtrace	5	140 µg Fe	Lymph node
	400	2800 µg Fe	Injection spot
^99m^Tc	5	≈ 5.75 MBq	Lymph node
	20	≈ 23 MBq	Injection spot

### Experiments

**Fig. 1:**
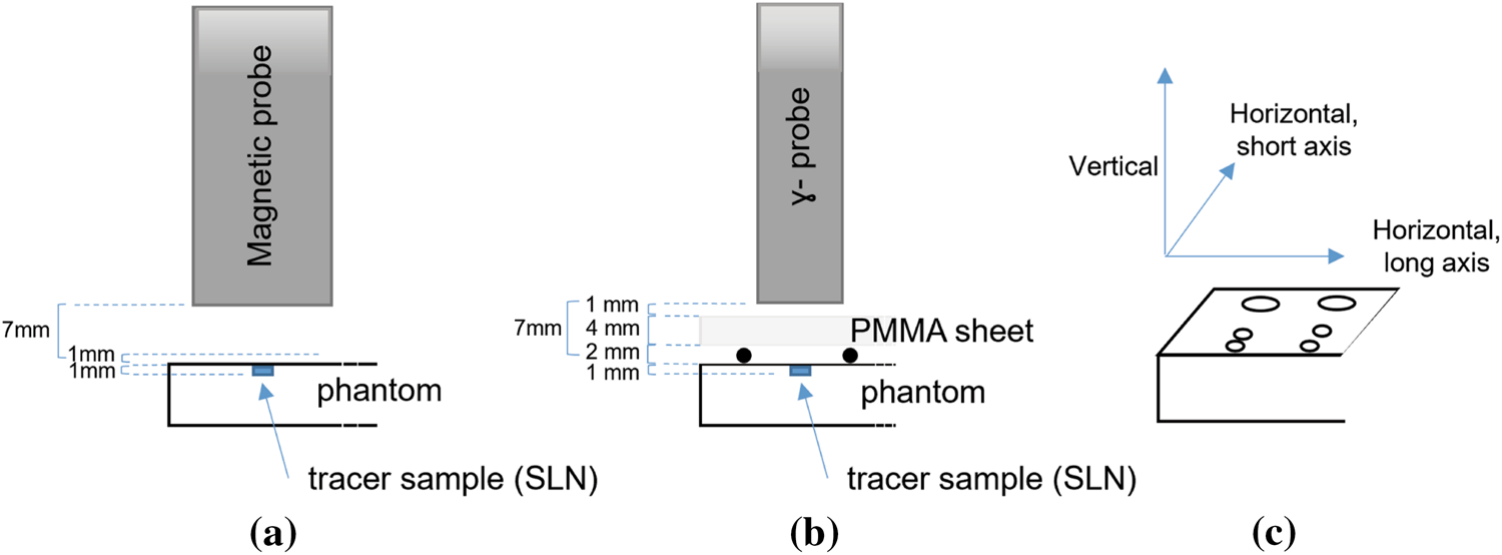
Schematic representation of setup, measuring a tracer sample. **a** Magnetic probe setup. **b** Gamma probe setup. Black dots between the phantom and polymethyl-methacrylate (PMMA) sheet represent PMMA supports, preventing the PMMA from touching the radioisotope sample. **c** Schematic representation of phantom with axis orientation

Figure 1 provides an overview of the experimental setup employed in this study. To carry out the experiments consistently, the probes were positioned with a robotic arm (Meca500-3R, Mecademic, Canada) and aligned in a vertical orientation relative to the phantom surface. This standardized arrangement ensured uniformity across all experimental procedures. All data processing was performed by software developed in-house (using MATLAB environment, Version 2021b, MathWorks Inc., Natick, Massachusetts, USA).

To enhance the reliability of magnetic detection and minimize false positive results, counts obtained when a sample was near the probe were deemed to be detections if they surpassed the background counts level by one standard deviation (the detection threshold). The background signal (bs) was the number of counts not changing by increasing the distance to the sample. For detection of the magnetic tracer, the data for both magnetic probes were acquired in triplet and used to calculate the average signal and standard deviation (SD). The average signal was normalized between 0 and 1. Prior to data acquisition, the detection threshold was calculated as defined by Eq. 1. This served as a reference point for assessing the presence of detectable signals.

1 $$\mathrm{threshold}=\mathrm{mean}\left(\mathrm{bs}\right)+(1\mathrm{SD}\left(\mathrm{bs}\right))$$

For detection of radioactive tracer, the phantom and probe were separated by an additional layer of polymethyl-methacrylate (PMMA) transparent thermoplastic.

#### Longitudinal Distance

**Fig. 2:**
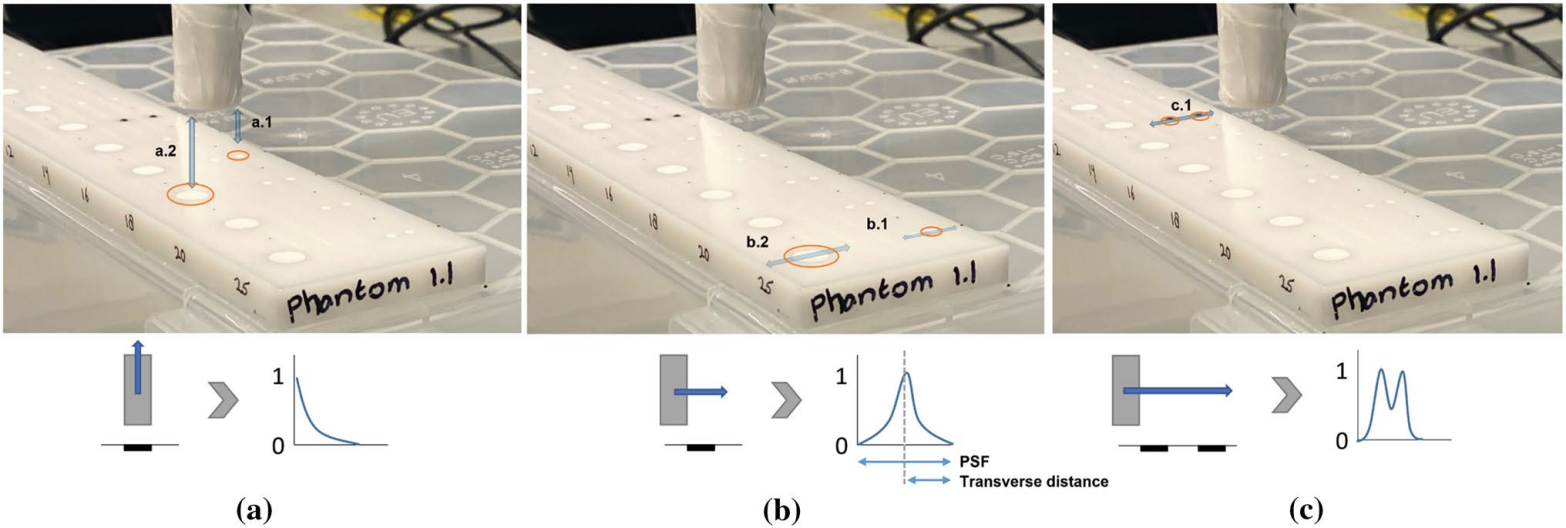
**a** Longitudinal distance evaluation, *a.1* measurement of an SLN and *a.2* of an injection spot. **b** Transverse measurements of individual tracer volumes for full width at half maximum (FWHM) calculation, and maximum transverse distance, *b.1* measurement of an SLN and *b.2* of an injection spot. **c** Transverse measurements for resolving power evaluation (*c.1*, two adjacent samples)

Defined as the maximum longitudinal distance along the long axis of the probe at which the probe can detect the tracer sample. Readout values of the probe, in counts, were collected for the small and large tracer volumes separately, starting with the probe tip at a distance of 1 mm for the magnetic probes and at 7 mm for the gamma probe. The data were acquired in an upwards interval movement with a step size of 1 mm for the magnetic probes. For the gamma probe, acquisition was performed with a step size of 5 mm (for the small tracer volume), and a step size of 10 mm (for the large tracer volume) (Fig. 2a). The longitudinal distance was established as the last measurable value above the detection threshold.

#### Transverse Distance

Defined as the maximum transverse distance, perpendicular to the long axis of the probe, at which the probe can detect the tracer sample. It was assessed for small and large tracer volumes separately. For this experiment, an individual pit in the phantom was filled with a tracer, followed by moving the probe horizontally across the phantom at a fixed vertical distance of 7 mm (Fig. 2b). The step size was set to 1 mm for magnetic probes and to 5 mm for the gamma probe. Transverse distance was established as the last measurable distance above the detection threshold.

#### Resolving Power

Defined as the shortest distance between two tracer samples that can be resolved. The acquired signal response to the sample point source was used to assess the resolving power. At the height corresponding to the full width at half maximum (FWHM), which is 50% of the highest acquired signal, we examined whether the peaks of the signal response acquired from the two individual point sources intersected. If the intersection of the peaks fell below the 50% threshold, we concluded that the probe could resolve each signal source. Additionally, data were acquired for two adjacent samples with a small tracer volume and the resolving power was evaluated. The probe was moved horizontally over the short axis of the phantom at 7 mm height from the phantom surface with a step size of 1 mm for magnetic probes and 2 mm for the gamma probe (Fig. 2c). For the DiffMag handheld probe, additional measurements were performed at 1 mm height from the phantom surface. A Gaussian mixture model (GMM) was used to smooth the data.

## Results

Table 2 summarizes the results for all three probes and for both tracer volumes: small and large. The results of the small and large tracer volumes are provided individually for each probe, involving longitudinal and transverse distance as well as the resolving power. All values are given in millimeters (mm). For a detailed view of the performance measurements see the sections “Longitudinal Distance”, “Transverse Distance”, and “Resolving Power”.

**Table 2:** Summarized performance for all three probes assessed at 7 mm distance to the phantom. The small tracer volume was magnetic tracer = 140 µg Fe, radioisotopes ≈ 5.75 MBq. The large tracer volume was magnetic tracer = 2800 µg Fe, radioisotopes ≈ 23 MBq)

Device/Tracer 1 SD	Longitudinal distance (mm)		Transverse distance (mm)		Resolving power (mm)	
	Small	Large	Small	Large	Small	Large
DiffMag/Magtrace	12	17	13	21	14	15
Sentimag/Magtrace	10	15	11	18	12	18
Gamma probe/^99m^Tc	>120	>170	51	55	18	25

### Longitudinal Distance

**Fig. 3:**
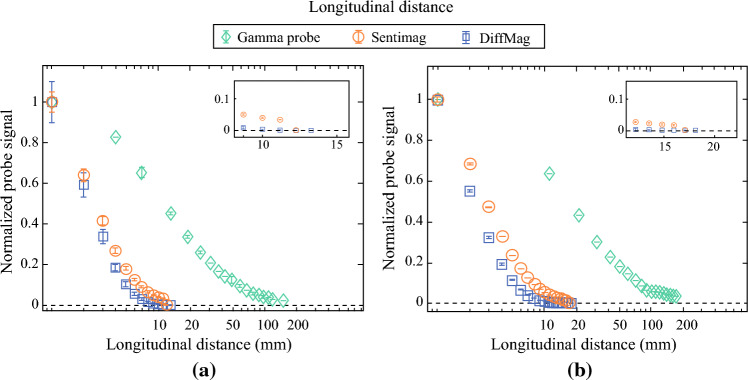
Longitudinal distance (mm) measured for 140 µg Fe (**a**) and 2800 µg Fe (**b**) Magtrace using DiffMag and Sentimag probes, and for ≈ 5.75 MBq (**a**) and ≈ 23 MBq (**b**) ^99m^Tc using gamma probe

The normalized graph as a function of longitudinal distance for all three handheld probes and the threshold, marked at 0, are presented in Fig. 3 for small and large tracer volumes. For the small tracer volume, longitudinal distances of 12 mm, 10 mm, and > 120 mm were observed for the DiffMag handheld, Sentimag, and gamma probe, respectively. For the large tracer volume, the longitudinal distances were: 17 mm, 15 mm, and > 170 mm, respectively.

### Transverse Distance

**Fig. 4:**
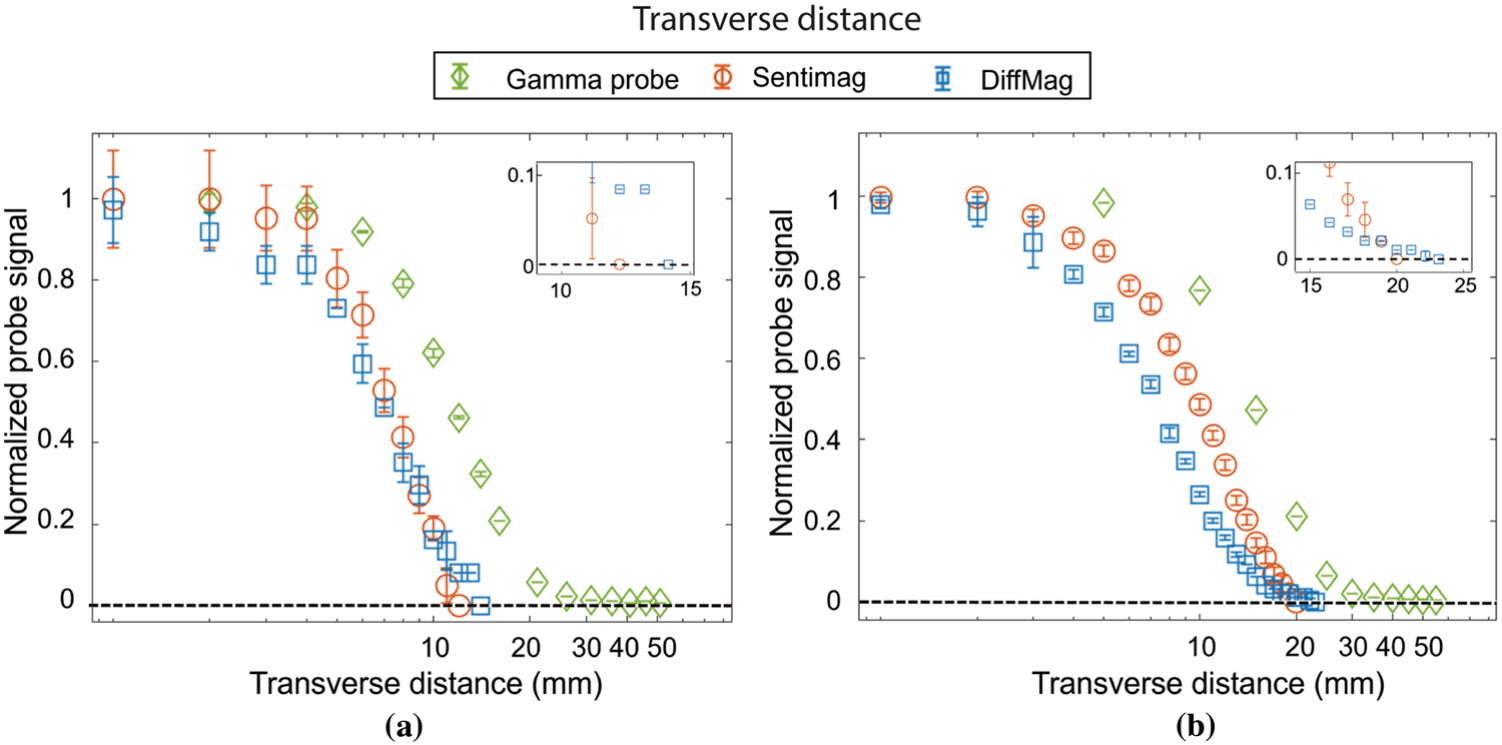
Transverse distance for DiffMag handheld, Sentimag, and gamma probe at 140 µg Fe/≈ 5.75 MBq (**a**) and 2800 µg Fe/≈ 23 MBq (**b**)

The normalized graph shown in Fig. 4 indicates the transverse distance for all three handheld probes. For the small tracer volumes, the transverse distances were 13, 11, and 51 mm for DiffMag handheld, Sentimag, and gamma probe, respectively. For the large tracer volumes it was 21, 18, and 55 mm, respectively.

### Resolving Power

At a height of 7 mm above the phantom surface, the FWHM of the small tracer volume for the DiffMag handheld, Sentimag, and gamma probe was established at 14 mm, 12 mm, and 18 mm, respectively. For the larger tracer volume these were 15 mm, 18 mm, and 25 mm, respectively.

**Fig. 5:**
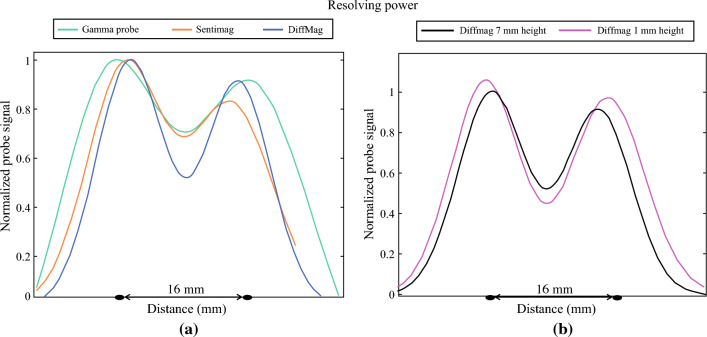
**a** Application of Gaussian mixture model on results of two adjacent small tracer volumes 16 mm apart, the different probes at 7 mm height from the phantom surface. **b** For the DiffMag handheld probe, two adjacent small tracer volumes 16 mm apart, measured at 1 mm and 7 mm height above the phantom surface

The GMM was applied to the raw data of two adjacent tracer volumes. Figure 5a, illustrates an example for all handheld probes: two small tracer samples (140 µg Fe or ± 5.75 MBq each) at 16 mm distance. Figure 5b, shows the DiffMag handheld probe measurements of two small tracer samples 16 mm apart at different heights (1 and 7 mm) to the phantom surface. This example shows that increasing the height of the DiffMag handheld probe above the sample leads to a decrease in resolving power.

## Discussion and Conclusion

In this study we assessed the effectiveness of the DiffMag handheld probe by using a clinically relevant phantom for FOM cancer. Specifically, we evaluated the performance in terms of LN position relative to the injection spot and the volume of tracer accumulating in each individual SLN. Regarding the longitudinal distance, the gamma probe detects the tracer at a distance that is 10 times greater than either magnetic probe. When comparing the DiffMag handheld probe with the Sentimag probe, it was observed that the Sentimag exhibited a higher number of counts at similar short distances. In a clinical context, the primary objective is to identify the SLN, making the actual count number less relevant. The relatively higher number of Sentimag counts may imply an easier differentiation between the SLN and background signal. However, the need for continual balancing during measurements potentially negates this advantage. Regarding the lateral sensitivity, the gamma probe had a 3–4 times wider reach than either magnetic probe, while the magnetic probes performed similarly. With respect to the resolving power, the results of the FWHM of the individual point source measurements and the phantom with two adjacent tracer volumes did not correspond. Where the magnetic probes should perform equally and better than the gamma probe, Sentimag did not seem to meet these expectations, but rather performed similar to the gamma probe. It should be noted that without the PMMA sheet, the FWHM of the gamma probe could be a bit smaller, due to less scattering, and could therefore have an even a higher resolving power. However, PMMA was added to mimic the clinical setting for the gamma probe, i.e., the gamma-radiation attenuation by tissue. In the case of magnetic measurements, addition of a PMMA sheet does not mimic the clinical setting and was therefore left out. The distance of the probe to the sample influences the resolving power. The DiffMag handheld probe showed less resolving power as the distance to the sample increased, meaning that the DiffMag handheld probe distinguished better when it approached the sample.

For the clinical requirements for SLNB in the head and neck region, a FWHM of 15 mm is required to distinguish two individual lymph nodes at 10 mm depth.^25^ Considering the high lateral sensitivity and resolving power established in this study, utilizing radioisotopes will not meet this requirement, leading to the disturbing shine-through phenomenon. Based on the FWHM, both magnetic probes seem promising for use in the head and neck region, especially in the FOM. However, this study does not demonstrate the effect of human body diamagnetism on the Sentimag signal, hence the clinical setting is not mimicked. To evaluate this influence in more detail and compare the results with the DiffMag handheld probe, a saline solution could be added to the experimental setup and closed samples should be used. The phantom was developed to investigate the performances of the different probes regarding clinically relevant distances in the FOM area. Besides this area, this phantom can be used to test the performance of other probes or tracers. Regarding the magnetic detection technique, this seems most suited for other clinical cases in which the injection spot and SLNs are in close proximity to each other. Given the fact that the DiffMag has several advantages over current Sentimag and gamma probes, it could be extended to other areas. The longitudinal distance of DiffMag is limited, which should be considered. We hypothesize that the DiffMag handheld probe would be more user friendly in a clinical setting compared with the Sentimag. Another limitation relates to the measurements with the gamma probe: the acquisitions were performed once, and the longitudinal distance was not measured. Since it already outperforms both magnetic probes by at least a factor of 10, the gamma probe is best suited for deeply located SLNs.

In conclusion, results of both magnetic probes were shown to be comparable in this setup, with the DiffMag handheld probe having the advantage of not needing balancing. Regarding the performance on longitudinal distance, the gamma probe reached 10 times deeper but had a lower resolving power than the DiffMag handheld probe. Thus, with a high resolving power but limited longitudinal distance, the DiffMag handheld probe was found suitable for further investigation when detecting SLNs in close proximity to the primary tumor, for example in the FOM.

## Contributions to Knowledge

This study adds insights into the performance of magnetic handheld probes compared with currently often utilized gamma probes.

The key implications with respect to public health involve informed decision making in case of unavailability of radioactive tracers.

The key implications with respect to clinical practice, when conducting SLNB, especially in the floor of the mouth, involve improved differentiation between the injection site and lymph nodes. It is thereby expected to decrease the shine-through phenomenon and improve the diagnostic outcome.

## Figures and Tables

**Fig. 6:**
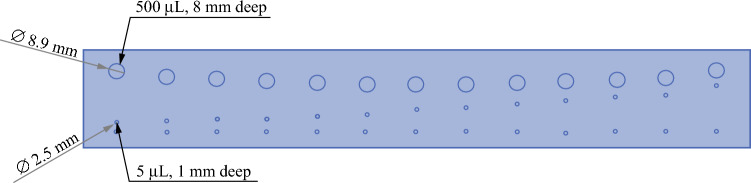
A Delrin^®^ phantom shows within each column one big pit (capacity 500 µl) representing one injection spot and two small pits (capacity 5 µl) representing the SLNs

## Appendix A

See Fig. 6.
